# What Is So Great about Inpatient Rehabilitation from the Patient Experience Perspective: Qualitative Content Analysis of an Appreciative Inquiry during a Bedside Experience Rounding

**DOI:** 10.3390/healthcare12171711

**Published:** 2024-08-27

**Authors:** Tiago S. Jesus, Julia Buschbacher, Jan Struhar, Taylor Walters, Courtney Lopez, Andrea Fernandez, Kristen Gracz, Karen Colby

**Affiliations:** 1 Division of Occupational Therapy, School of Health and Rehabilitation Sciences, College of Medicine, The Ohio State University, Columbus, OH 43210, USA; julia.buschbacher@osumc.edu (J.B.); taylor.walters@osumc.edu (T.W.); 2 Shirley Ryan AbilityLab, Chicago, IL 60611, USA; jstruhar@sralab.org (J.S.); clopez03@sralab.org (C.L.); afernand03@sralab.org (A.F.); graczkristen@gmail.com (K.G.); kcolby@sralab.org (K.C.)

**Keywords:** patient experience, patient-centered care, patient perspective, bedside rounding, content analysis

## Abstract

Background: Positive person-centered attributes of inpatient rehabilitation need to be identified from the patient’s perspective to be further developed and sustained. Purpose: To identify which attributes patients openly evoke as being great care experiences, using an open appreciative inquiry during the inpatient rehabilitation stay. Methods: Qualitative secondary analysis of appreciative patient comments during a bedside patient experience rounding facilitated by a neutral party was performed. Two independent analysts employed an inductive, summative form of content analysis. Results: Among 150 patients rounded, 122 provided categorizable appreciative accounts. Over two-thirds of the patients (67.2%) focused on “*staff attributes*” in their great-experience accounts. Those attributes were mostly interpersonal such as being “*attentive & caring—beyond clinical duty*” and being “*encouraging (but not too hard) & reassuring*”. These interpersonal staff attributes were reported with words showing deep levels of personal significance or patient appreciation. Beyond staff attributes, the perceived quality of “*patient care*” (31.1%) and opportunities for “*leisure and social activities*” (9.0%) were also frequently evoked. Amenities like *food or customer service* were the least evoked, rarely so as an exclusive attribute (0.8% for each). Conclusions: The human(e) factor, especially the interpersonal qualities of staff, emerged as greatly appreciated from the patient experience perspective during inpatient rehabilitation. These experiences help identify which person-centered attributes of care might be further developed and sustained.

## 1. Introduction

The *patient experience* of care is part of the quality and value of healthcare [1,2]. This experience refers to how patients experience and appraise valuable aspects of healthcare, such as staff communication, involvement in decision-making, or preparation for discharge, among other key interactions with health providers [3]. For broader healthcare, systematic reviews have associated better patient experience scores with better treatment compliance, better patient-reported physical and mental health, and finally, lower healthcare utilization [4,5,6,7]. In the rehabilitation field, patient-centered care approaches (e.g., collaborative goal setting) have been, for example, associated with greater psychosocial outcomes (e.g., self-efficacy, emotional status, quality of life) [8,9,10,11].

Patient experiences can be systematically elicited, processed, and disseminated (e.g., relayed to providers; publicly reported) as one way to monitor the *person-centeredness* of care as well as for informing patient-centric quality improvement (QI) activities [3,12,13]. However, a recent scoping review of the rehabilitation literature found a dearth of studies using patient experience feedback for service redesign and QI activities [14]. Among those studies, none used patient experiences at the point of care, either to timely inform immediate service recovery or to positively affect the patient experience while patients are receiving care (e.g., during the inpatient stay) [14]. Patient experience data are often obtained through post-discharge, externally collected surveys, which leads to relatively low response rates (e.g., 20–30%), recall bias, and often from non-representative patient samples [15,16]. Post-discharge experience survey data are valuable for benchmarking and large-scale data analysis.

For instance, within inpatient rehabilitation facilities (IRFs), standardized post-discharge patient experience data helped to identify which factors (e.g., personal issues) most contribute to better overall patient experiences, but at a response rate of 16.2% [17]. For person-centered QI purposes, these data benefit from the complement of other, more personalized forms of feedback (e.g., narratives, photovoice, open qualitative comments) [18,19], including at the point of care (e.g., during inpatient stay), while being inclusive of a wider serviced population [15,20,21]. Of note, rehabilitation services are best described by their aim to restore or return a person to a state of optimal functioning in interaction with his or her environment, which goes over and beyond any underlying medical diagnosis generating a functional impairment [22]. In turn, inpatient rehabilitation refers to a more intensive form of rehabilitation, often involving multiple therapeutic disciplines; for example, in the US, inpatient rehabilitation units provide at least three hours of intense rehabilitation services (e.g., three hours of therapy) per day of inpatient stay, typically after acute-care hospitalizations [23].

In the context above, one large freestanding facility in the US developed and pilot implemented a new process to elicit near real-time patient experience feedback, designed to be then systematically relayed to providers—hence, capable of enabling immediate, patient-centric service recovery. Following guidance from systematically reviewed evidence [24], this process included an in-person, mixed-methods bedside experience rounding conducted by a trained neutral party who digitally inputted both the quantitative and qualitative patient experience data. Incorporating appreciative inquiry tenets [14,25,26], that in-person patient experience rounding included an initial open question for each patient to describe a great care experience they had during their inpatient stay and what they wished to describe. 

In the rehabilitation literature, common ‘negative’ experiences during inpatient rehabilitation stays (e.g., disempowerment, boredom) have been described [27,28], but we know much less from the appreciative standpoint (i.e., ‘positive’ patient experiences as identified by patients themselves during their inpatient stays). These ‘positive’ experiences are the focus of this paper. Identifying those ‘positive’ experiences in close proximity to care delivery can help build the knowledge of what is valued by patients within the attributes of inpatient rehabilitation services. Understanding these person-centered attributes should help inform what should be developed, prioritized, sustained, or rewarded in rehabilitation practice from the ‘positive’ patient-experience perspective.

In the context above, using existing data from a previously tested in-person bedside experience rounding, we aim to respond to the following study question:

- Which are the ‘positive’ or great experiences of care that patients often evoke at the point of care (i.e., during inpatient rehabilitation stay) when openly elicited from an appreciative standpoint?

Overall, identifying these typical ‘positive’ experiences will highlight which attributes of care are most valued by patients and might be reinforced in inpatient rehabilitation practices. This appreciative, strengths-based inquiry [25,26] differs from identifying typical ‘negative’ experiences to be mitigated—with the latter more widely known from the literature [27,28].

## 2. Methods

Design: A secondary, external analysis of anonymized qualitative patient comments from an in-person bedside experience rounding was performed. A secondary analysis of qualitative data involves the re-use of pre-existing qualitative data to investigate a new or additional research question that can be answered by an existing dataset [29]. In other words, it refers to the use of qualitative data collected by someone else or to answer a different research question [30].

Here, the secondary qualitative analysis was primarily conducted by external, university-based researchers over a dataset generated by QI activity at one inpatient rehabilitation facility: Shirley Ryan AbilityLab. The QI activity, on a bedside experience rounding, primarily involved eliciting and using ‘negative’, posit-of-care patient experiences which could be used for immediate QI purposes; the process evaluation of that primary goal (initial feasibility and utility of that QI activity) was published elsewhere [31]. However, the data obtained during that QI activity also involved an inquiry about ‘positive’ patient experiences during the inpatient rehabilitation stay. That dataset of ‘positive’ experiences was not used for knowledge-building purposes before. Here, that previously collected dataset was a posteriori, secondarily analyzed by external researchers toward responding to this study’s question set a posteriori, which are the typical great experiences of care, as reported by patients themselves during inpatient rehabilitation.

In line with a secondary data analysis [29,30], the anonymized patient comments were qualitatively analyzed by external researchers who had no direct contact with any of the patients. For the analytical approach, we used an inductive-based, summative form of qualitative content analysis, which is focused on counting the frequency of specific words or latent content as a mechanism of no extrapolation of meaning but of identifying and quantifying usage of content emerging from the data [32,33].

As a QI activity with secondary analysis of anonymized data, it does not meet the criteria for human subject research. We used the SQUIRE guidelines for reporting QI studies [34] and the SPQR guidelines for qualitative studies [35].

Setting: The QI activity took place from February to September of 2023 in a large, freestanding IRF in the Midwestern USA (Shirley Ryan AbilityLab). The Shirley Ryan AbilityLab is a state-of-the-art translational rehabilitation research hospital in downtown Chicago dedicated to advancing human ability through innovative rehabilitation and research. The facility has contemporary architecture, equipment, service delivery concept, layout, and technological environment, and has been ranked the no. 1 rehabilitation hospital in the USA for over three decades [36]. These factors may affect the patient experience of care and the inpatient stay, either in a positive direction (e.g., by the experience of state-of-the-art facilities and model of care) or in a negative direction (e.g., high-bar a priori expectations to obtain nothing less than excellent care, services and experiences which may lead to easier frustration when some experience is perceived as suboptimal). Specifically, the QI activity occurred within a unit focused on strengthening and endurance, i.e., not diagnostic-focused.

Data collection: The data for this study were derived from a QI activity, which referred to the introduction of bedside experience rounding to elicit patient experiences of what was great during their length of stay in a timely manner. The resultant data were then relayed to the service-unit leaders for individual-level corrective action as needed. In short, the goal was to follow person-centered care tenets toward eliciting and then acting upon personalized patient experience feedback [31]. While the previous study was focused on a process evaluation of the implementation of the whole process, which was found acceptable and usable by both staff and patients (e.g., capable of reverting suboptimal experiences) [31], here we focus on the analysis of the experiences that patients reported as great, following appreciative inquiry tenets [25,26].

Specifically, the data collection took place during the experience rounding, facilitated by trained non-attending staff or hospital volunteers. As those rounders were not directly involved in the given patient’s care, these rounders were deemed as sufficiently ‘neutral’ parties to collect the patient experience feedback. These experience rounds occurred shortly before the patients reached their mid length of stay. When asking for great experiences, the rounders encouraged patients to freely share any positive experiences they had encountered thus far without being directed to any domain of the patient experiences (e.g., staff communication, amenities). This invitation for positive feedback was given before posing five directed questions regarding suboptimal experiences or suggestions for improvement. During the appreciative phase, rounders actively listened to the patient’s responses and immediately inputted them into a digital platform created for this purpose. Patients had the opportunity to view, listen to, and revise the typed content as needed. Before being sent externally for analysis, the data were internally de-identified, including blinding of staff names.

Participants: Throughout the pilot, all English-speaking patients without major cognitive or communication impairments serviced by the unit over the 8-month period were included. That means we had no sampling procedures beyond the abovementioned categorial exclusions. Plans for a second phase to adapt the process for the entire patient population exist based on the positive appraisal of the initial pilot. During the 8-month implementation, all 150 eligible patients were approached and asked an appreciative question. Participants could decline or omit specific experiences.

The types of patients serviced by the inpatient rehabilitation unit subject to the QI activity are those with functional needs for “strengthening and endurance”. The impairment in these functions arises from non-restricted diagnosis (e.g., patients with deconditioning after major surgeries and patients recovering from debilitating cancer treatments). This focus on impairments and functional needs, over and beyond underlying diagnoses, is one of the innovative features of the organization of the Shirley Ryan AbilityLab as a leading translational rehabilitation hospital.

Analysis: We conducted an inductive summative qualitative content analysis [32,33]. The summative content analysis differs from a conventional content analysis as it is primarily based on a count, frequency, or sum of keywords or content as a step toward synthesis or interpretation, with no attempt to identify underlying meanings [32,33]. Within this methodology, we used an inductive approach as opposed to a directed form of content analysis (or even a framework synthesis [37,38] or template analysis [39]), which are deductive approaches in which the initial codes are derived from a previous framework, research findings, or theory. In short, our goal was to identify emerging attributes from the data without imposing preconceived frameworks. Of note, one of the analysts previously published models on person-centered rehabilitation [40] and rehabilitation quality of care [41]; however, no attempt was made to fit the data.

Each patient quote was categorized, excluding generic (e.g., “all was great”) or ambiguous statements (“great atmosphere” could be about the physical or social environment). Two external researchers performed initial open coding and axial coding independently. Disagreements were resolved through discussion. To enhance trustworthiness, a third independent analyst analyzed the results. Final changes were made based on this process, with three patient comments recategorized. The final coding structure used is provided in Supplementary Table S1.

## 3. Results

Within the results, we provide (1) a numerical overview of the participant comments, (2) the major categories emerging from the analysis, and finally, (3) subcategories of staff attributes as the most frequently reported category of great patient experiences.

1. Numerical overview

A total of 136 of 150 patients (90.6%) reported positive remarks about their experience of care. Among them, 122 (89.7%) provided classifiable accounts—the *corpus* under analysis.

Among those 122 patients, 70.5% (*n* = 86) evoked only one category for their great-experience accounts; the remaining provided comments fitting into two or three categories. No patient provided comments that fit into four or more analytical categories.

2. Major categories from the content analysis

Table 1 shows the frequency of patient comments per major category/attributes reported. Over two-thirds of the patients (*n* = 82; 67.2%) focused on *staff attributes* (e.g., interpersonal, professionalism), 58 of them exclusively so (44.3% of the total). In a later results section, we detail subcategories and illustrative quotes. The *patient care* process (e.g., perceived quality or experience of progress) was the second most evoked category (*n* = 40; 32.5%), inclusive of 24 mentions exclusively so (19.5% of the total).

No other categories had more than 10% of specific mentions (Table 1). Among them, *leisure and social activities* were the most common. Patients reported that they appreciated the “*group therapy and [the] chance to meet new people*” (patient #6) and alluded to the benefits: “*it helps me mentally. I like socializing with other patients*” (patient #36). Other social, outdoor, or recreational opportunities in the garden, cafeteria, or outside of the facility were mentioned for the downtime. The *built environment*, including nice views, and *teamwork,* were also evoked from the appreciative patient experience perspective. For example, one patient noted that “*you can tell there is communication between the team*” (patient #44).

Specific amenities (e.g., food service, customer service, cleanliness) were other mentions, but with one exclusive mention each and less detailed accounts; however, if combined, these amenities would be the third most evoked category.

3. Subcategories of staff attributes

Table 2 reports subcategories of staff attributes evoked; the initial four are on interpersonal attributes.

Being *attentive and caring—beyond clinical duty* was the most frequent category, with 25 mentions, 16 of them just for this attribute. Illustrative comments are below:

*“I think they have gone beyond their duties to make me feel comfortable”*, patient #3.

*“I find the staff is paying attention to my care with things I don’t even speak up about. My foot was drooping to the side and the nurse noticed and got me boots to help. I didn’t even ‘aask’ her [emphasis]”*, patient #24.

Being *kind, friendly, and smiling* was also frequently reported (*n* = 16), although only six times exclusively. The keyword “*friendly*” was common, along with mentions of kindness or smiling faces, albeit without detailed patient accounts.

Ten patients alluded to staff members being *encouraging (but not too hard) & reassuring*, nine exclusively so. The delicate balance of providing such encouragement is illustrated below, sometimes with notes of deep appreciation:

*“I was pushed but not forced, it wasn’t like a boot camp experience”*, patient #94.

*“They reassure me where I am at. If I am having a lousy day they don’t push, they listen and are there but don’t push me”*, patient #46.

*“[She] toldddddd [emphasis] me to give it some time and see how everything works and if I still didn’t like it then go home. She was right! My stay here has been life-changing and I am appreciative”*, patient #100.

*“[He/She] tells me I am doing good and beginning to walk, that’s a blessing to me; they inspire you to do good”*

*“The encouragement I receive from everyone especially my [therapists], I just love them. It has become personal when I make accomplishments; I am wanting to make them proud and they celebrate with me and make me feel special”*, patient #107.

Patients also appreciated when staff were *listening & responsive to* their needs or questions, with 10 total mentions, five exclusively so. This also involved adaptive communication and care adjustments:

*“The way they take your time and teach me. They adapt to their patients”*, patient #87.

*“[She] is wonderful and such a great listener, if I have a problem she listens and tries to fix it. When she walks out the door, I never feel like something was unresolved”*, patient #12.

*“Staff listen to me and they make adjustments to my preference”*, patient #109.

Eight comments (one in exclusivity) focused on staff being *professional & knowledgeable*, intersecting their politeness and expertise. Finally, 28 patients provided comments appreciating staff but were not subcategorizable.

## 4. Discussion

Under an appreciative approach, this study identifies what patients frequently and openly evoke as ‘great’ experiences during inpatient rehabilitation. We employed a strengths-based approach to identify “what is going well” for that to be strengthened, replicated, reinforced, rewarded, and/or sustained in practice. That *positive* data might complement “what is going wrong” and thereby may require corrective action [25,26,42]. The patients’ illustrative quotes, especially related to staff interpersonal attributes, also showed deep levels of gratitude and appreciation. Once collected and relayed to staff in a timely manner, these accounts may be useful for staff engagement, recognition, and behavioral reinforcement.

Our findings highlight a notable emphasis on staff interpersonal and caring attributes, surpassing patient care and facility attributes. That partly aligns with recent *big data* analysis of post-discharge patient experience data in IRFs [17], using data from a 10-year period, albeit based on a response rate of 16.2%. For instance, Park and colleagues’ analysis of post-discharge surveys suggests that a willingness to recommend the hospital is mostly determined by personal issues, such as communication with staff, pain control, and communication upon arrival [17]. These determinants were followed by physical therapy care, occupational therapy care, nursing care, discharge, and rehabilitation doctor care [17]. While overall aligned, our qualitative analysis provides a complementary dataset, with a point-of-care snapshot of patient-evoked great experiences providing illustrative accounts, especially among staff interpersonal attributes. Detailed patient accounts can be more instrumental for improvement purposes, complementing standardized post-discharge data, and are often more useful for benchmarking purposes [18,19]. 

Beyond frequently evoked, the interpersonal attributes of staff emphasized specific provider behaviors. Compared to subjective positive appraisals, specified behaviors are more easily replicable, reinforced, or enacted either through data relay to providers [31] or broader scientific reporting [40,43]. Other categories evoked by rank order were the perceived quality of the patient care process, opportunities for leisure and social activities, the built environment, and teamwork. Specific amenities were less frequently evoked and rarely exclusively so. These seemed ‘nice to have’ but often less fundamental than the human(e) side of care.

Among interpersonal attributes, being *attentive and caring—beyond clinical duty* was the most frequently mentioned by the patients, emphasizing the staff’s genuine interest and caring attitude. With that shown attitude, patients were likely experiencing being personally valued and cared for [40,43,44]. Patients’ comments also focused on staff being *encouraging (but not too hard) and reassuring*; when this attribute was evoked, it was often exclusively so and with deep appreciation reported. The rehabilitation literature has long grappled with the delicate balance of encouraging patients without pushing too hard, a balance that includes motivating without reinforcing unrealistic expectations [45,46]. The literature has also emphasized the need to be adaptive to the situation at hand. This involves observing patient cues to discern when to push and when to stop and instead acknowledge and reassure [40,47]. One could hypothesize that in these reported situations, staff members adeptly navigated a complex balance, seamlessly transitioning from being encouraging to reassuring and vice versa.

In the literature, one can find some typified negative experiences in IRFs (e.g., boredom, lack of activity in the downtime) [27,28,48]; here, we found some symmetrical experiences on the positive side, such as joy with social activities, leisure, or engaging with peers. These findings perhaps reinforce the importance of IRFs creating opportunities for such experiences. Socialization, in addition to being valuable for many patients, can be facilitated by the environment [49]; here, socialization opportunities were partly linked to the environment (e.g., having a garden or cafeteria) or to group-based therapy. Previous studies have demonstrated that it is possible to create and engage patients in creating opportunities for adding social and leisure opportunities when those were not present by design [48,50].

Regarding the staff’s teamwork, features such as shared communication and care coordination also emerged in the great-experience accounts. While patients may not directly observe coordinating activities, they can firsthand experience whether the care or messages delivered to them are aligned, articulated, or congruent with one another [40,41,51].

Importantly, patients responded to the same prompt within the same IRF, with a broad spectrum of attributes that they individually experienced as great. Patients are individuals with their own set of values, preferences, lived experiences, and other human factors that make them and their experiences personal and unique. We follow the standpoint that there is a *person* beyond the *patient* and that the one-size-fits-all approach applies toward operationalizing a person-centered rehabilitation or great patient experiences [40,52,53]. Hence, the timely elicitation and relay of appreciative feedback (along with any feedback for corrective action) can be a key mechanism for a personalized, person-centered use of each patient experience’s feedback for QI purposes. In turn, patients involved in the process evaluation of this QI activity appreciated having the opportunity and a mechanism to express and convey their gratitude to staff on their own terms [31].

Overall, this study shows which attributes of care are more frequently evoked as great experiences of care from the patient experience perspective. Staff interpersonal qualities stood out among what was so greatly appreciated from the patient experience perspective, for example, in contrast with amenities. Service managers and clinicians might become aware of these and other experiences that their patients value, either typically or as individuals with unique preferences (e.g., elicited by bedside rounding mechanisms) [24,31]. Then, service managers or QI champions can develop in-service training or other QI activities for services and frontline practitioners to further develop or reinforce their capacity to promote a ‘positive’ patient experience with care, as typically evoked by their own patients [14]. Finally, for staff engagement, recognition, and behavioral reinforcement, it may be important to create formal mechanisms to timely relay positive experience data to both frontline providers and their service-unit leadership [25,31].

### Limitations

The study had the following limitations. First, the data were collected from a single facility and one unit, albeit multi-diagnostic. Features of the facility (e.g., new architecture, high-tech environment, high-rise views, widely known reputation) can affect the patient experience or the patient’s expectations for that experience. Hence, the results here, such as frequency distribution, may not be representative of other service delivery contexts. Second, appreciative inquiry tenets were not a full appreciative inquiry approach toward strengths-based, client-engaged service [25]. Third, the analysis was focused on openly evoked positive experiences purposely biased toward *positive deviant* experiences [43,54]; those cannot be understood as the whole, balanced experience of the inpatient stay. Fourth, the secondary external analysis contributed to the analytical independence but implied that cues from the patients were not observed or interpreted during data collection beyond the rounders’ input, such as elongated wording for emphasis. Finally, while we included the vast majority of patients serviced by the unit during the pilot, the rounding under testing was not, at this stage, inclusive of the experiences of people with severe cognitive or communication impairments or of those unable to speak in English. Those experiences can be qualitatively different and are planned to be accommodated into future QI cycles.

## 5. Conclusions

The human(e) factor, especially staff interpersonal qualities, stood out among what was so greatly appreciated from the patient experience perspective during inpatient rehabilitation stays. Among these attributes, being “attentive & caring—beyond clinical duty” stood out as an interpersonal quality most frequently evoked. These individual-level, preference-sensitive positive experiences can be timely elicited and rapidly relayed to providers for behavioral reinforcement and for patients to show gratitude and appreciation in their own words. As most patients identified interpersonal attributes as leading to a great experience, services and providers may need to prioritize the development of in-service training or development activities that emphasize these attributes that are so valued from a patient experience perspective.

## Figures and Tables

**Table 1:** Frequency and percentage of the patient comments per major category of analysis. We report the “total” number and percentage of patients whose comments fit that category, but also the number and percentage of patients whose comments were “exclusive” to that category (i.e., patients provided comments that only pointed to or emphasized that category alone).

Categories	Examples of Attributes	Total (*n*; %)	Exclusive (*n*; %)
Staff Attributes	Interpersonal attributes and behaviors; knowledge; professionalism	82; 67.2%	54; 44.3%
Patient care	Perceived quality and progress; therapy is great, enjoyable, varied, or spaced out	38; 31.1%	22; 18.0%
Leisure and social activities	Going outside; meeting new people; having fun with peers—including in group therapy	11: 9.0%	3; 2.5%
Built environment	Facility; pleasant physical environment; views.	5; 4.1%	2; 1.6%
Teamwork	Perceived alignment; shared information	5; 4.1%	2; 1.6%
Food service		6; 4.9%	1; 0.8%
Cleanliness		6; 4.9%	1; 0.8%
Bed quality		2; 1.6%	1; 0.8%
Customer service		1; 0.8%	1; 0.8%
Quietness		1; 0.8%	1; 0.8%
Equipment (gym)		4; 3.3%	0; 0%
Room quality		2; 2.4%	0; 0%

**Table 2:** Subcategories of staff attributes as reported by patients (*n* = 86), ordered by prevalence, among those with specific staff attributes or behaviors reported. We report the “total” number and percentage of patients whose comments fit that subcategory, but also the number and percentage of patients whose comments were “exclusive” to that subcategory (i.e., patients provided comments that only pointed to or emphasized this subcategory alone).

Subcategories of *Staff Attributes*	Total (*n*; % of Total)	Exclusive Subcategory (*n*; % of Total)
Attentive & caring—beyond clinical duty	25; 20.5%	16; 11.5%
Kind, friendly, and smiling	16; 13.1%	6; 4.9%
Encouraging (but not too hard) & reassuring	10; 8.2%	9; 7.4%
Listening and responsive to	10; 8.2%	5; 4.1%
Professional & knowledgeable	8; 6.6%	1; 0.8%
Overall positive appraisals of staff, i.e., not subcategorized	28; 23%	10; 8.2%

## Data Availability

Data are contained within the article and can be further obtained from the corresponding author under reasonable request.

## Supplementary Materials

The following supporting information can be downloaded at: https://www.mdpi.com/article/10.3390/healthcare12171711/s1, Table S1: Coding structure.

## Abbreviations

IRF	Inpatient Rehabilitation Facility
QI	Quality Improvement

## References

[1] Institute of Medicine (2001). Crossing the Quality Chasm: A New Health System for the 21st Century.

[2] National Quality Forum (2020). The Care We Need: Driving Better Outcomes for People and Communities.

[3] Agency for Healthcare Research and Quality What Is Patient Experience? Rockville, MD. https://www.ahrq.gov/cahps/about-cahps/patient-experience/index.html.

[4] Navarro S., Ochoa C.Y., Chan E., Du S., Farias A.J. (2021). Will Improvements in Patient Experience with Care Impact Clinical and Quality of Care Outcomes?: A Systematic Review. Med. Care.

[5] Goldfarb M.J., Bibas L., Bartlett V., Jones H., Khan N. (2017). Outcomes of Patient- and Family-Centered Care Interventions in the ICU: A Systematic Review and Meta-Analysis. Crit. Care Med..

[6] Park M., Giap T.T., Lee M., Jeong H., Jeong M., Go Y. (2018). Patient- and family-centered care interventions for improving the quality of health care: A review of systematic reviews. Int. J. Nurs. Stud..

[7] Rathert C., Wyrwich M.D., Boren S.A. (2013). Patient-centered care and outcomes: A systematic review of the literature. Med. Care Res. Rev..

[8] Rose A., Rosewilliam S., Soundy A. (2017). Shared decision making within goal setting in rehabilitation settings: A systematic review. Patient Educ. Couns..

[9] Yun D., Choi J. (2019). Person-centered rehabilitation care and outcomes: A systematic literature review. Int. J. Nurs. Stud..

[10] Levack W.M., Weatherall M., Hay-Smith E.J., Dean S.G., McPherson K., Siegert R.J. (2015). Goal setting and strategies to enhance goal pursuit for adults with acquired disability participating in rehabilitation. Cochrane Database Syst. Rev..

[11] Jesus T.S., Silva I.L. (2016). Toward an evidence-based patient-provider communication in rehabilitation: Linking communication elements to better rehabilitation outcomes. Clin. Rehabil..

[12] Berwick D.M. (2002). A user’s manual for the IOM’s ‘Quality Chasm’ report. Health Aff..

[13] Nundy S., Cooper L.A., Mate K.S. (2022). The Quintuple Aim for Health Care Improvement: A New Imperative to Advance Health Equity. JAMA.

[14] Jesus T.S., Stern B.Z., Struhar J., Deutsch A., Heinemann A.W. (2023). The use of patient experience feedback in rehabilitation quality improvement and codesign activities: Scoping review of the literature. Clin. Rehabil..

[15] Boissy A. (2020). Getting to Patient-Centered Care in a Post–COVID-19 Digital World: A Proposal for Novel Surveys, Methodology, and Patient Experience Maturity Assessment. NEJM Catal..

[16] Mercier M.R., Galivanche A.R., David W.B., Malpani R., Pathak N., Hilibrand A.S., Rubin L.E., Grauer J.N. (2021). Hospital Consumer Assessment of Healthcare Providers and Systems survey response rates are significantly affected by patient characteristics and postoperative outcomes for patients undergoing primary total knee arthroplasty. PLoS ONE.

[17] Park S., Xu J., Manes M.R., Carrier A., Osborne R. (2023). What Determinants Affect Inpatient Satisfaction in a Post-Acute Care Rehabilitation Hospital?. Arch. Phys. Med. Rehabil..

[18] Grob R., Schlesinger M., Barre L.R., Bardach N., Lagu T., Shaller D., Parker A.M., Martino S.C., Finucane M.L., Cerully J.L. (2019). What Words Convey: The Potential for Patient Narratives to Inform Quality Improvement. Milbank Q..

[19] Balbale S.N., Locatelli S.M., LaVela S.L. (2016). Through Their Eyes: Lessons Learned Using Participatory Methods in Health Care Quality Improvement Projects. Qual. Health Res..

[20] Jesus T.S., Papadimitriou C., Pinho C.S., Hoenig H. (2018). Key Characteristics of Rehabilitation Quality Improvement Publications: Scoping Review From 2010 to 2016. Arch. Phys. Med. Rehabil..

[21] Graham C., Käsbauer S., Cooper R., King J., Sizmur S., Jenkinson C., Kelly L. (2018). An evaluation of a near real-time survey for improving patients’ experiences of the relational aspects of care: A mixed-methods evaluation. Health Serv. Deliv. Res..

[22] Meyer T., Gutenbrunner C., Kiekens C., Skempes D., Melvin J.L., Schedler K., Imamura M., Stucki G. (2014). ISPRM discussion paper: Proposing a conceptual description of health-related rehabilitation services. J. Rehabil. Med..

[23] Centers for Medicare & Medicaid Services Inpatient Rehabilitation Facilities 2024. https://www.cms.gov/medicare/health-safety-standards/certification-compliance/inpatient-rehabilitation-facilities#:~:text=IRFs%20are%20free%20standing%20rehabilitation,intense%20rehabilitation%20services%20per%20day.

[24] Jesus T.S., Struhar J., Zhang M., Lee D., Stern B.Z., Heinemann A.W., Jordan N., Deutsch A. (2024). Near real-time patient experience feedback with data relay to providers: A systematic review of its effectiveness. Int. J. Qual. Health Care.

[25] Trajkovski S., Schmied V., Vickers M., Jackson D. (2013). Using appreciative inquiry to transform health care. Contemp. Nurse.

[26] Sandars J., Murdoch-Eaton D. (2017). Appreciative inquiry in medical education. Med. Teach..

[27] Luker J., Lynch E., Bernhardsson S., Bennett L., Bernhardt J. (2015). Stroke Survivors’ Experiences of Physical Rehabilitation: A Systematic Review of Qualitative Studies. Arch. Phys. Med. Rehabil..

[28] Kenah K., Bernhardt J., Cumming T., Spratt N., Luker J., Janssen H. (2018). Boredom in patients with acquired brain injuries during inpatient rehabilitation: A scoping review. Disabil. Rehabil..

[29] Heaton J. (2008). Secondary analysis of qualitative data: An overview. Hist. Soc. Res..

[30] Tate J.A., Happ M.B. (2018). Qualitative Secondary Analysis: A Case Exemplar. J. Pediatr. Health Care.

[31] Struhar J.G.K., Howe T., Sheth M., Fernandez A., Lopez C., Jesus T.S. (2024). Implementing a near real-time patient experience feedback process in inpatient rehabilitation: Mixed-methods process evaluation informed by the Normalization Process Theory. Int. J. Health Plann. Manag..

[32] Hsieh H.F., Shannon S.E. (2005). Three approaches to qualitative content analysis. Qual. Health Res..

[33] Kondracki N.L., Wellman N.S., Amundson D.R. (2002). Content analysis: Review of methods and their applications in nutrition education. J. Nutr. Educ. Behav..

[34] Ogrinc G., Davies L., Goodman D., Batalden P., Davidoff F., Stevens D. (2016). SQUIRE 2.0 (Standards for Quality Improvement Reporting Excellence): Revised publication guidelines from a detailed consensus process. BMJ Qual. Saf..

[35] O’Brien B.C., Harris I.B., Beckman T.J., Reed D.A., Cook D.A. (2014). Standards for reporting qualitative research: A synthesis of recommendations. Acad. Med..

[36] AbilityLab SR Shirley Ryan AbilityLab Ranked No. 1 by U.S. News & World Report for 33rd Consecutive Year 2023. https://www.sralab.org/articles/press-release/shirley-ryan-abilitylab-ranked-no-1-us-news-world-report-33rd-consecutive-year.

[37] Gale N.K., Heath G., Cameron E., Rashid S., Redwood S. (2013). Using the framework method for the analysis of qualitative data in multi-disciplinary health research. BMC Med. Res. Methodol..

[38] Russo G., Silva T.J., Gassasse Z., Filippon J., Rotulo A., Kondilis E. (2021). The impact of economic recessions on health workers: A systematic review and best-fit framework synthesis of the evidence from the last 50 years. Health Policy Plan..

[39] Brooks J., McCluskey S., Turley E., King N. (2015). The Utility of Template Analysis in Qualitative Psychology Research. Qual. Res. Psychol..

[40] Jesus T.S., Papadimitriou C., Bright F.A., Kayes N.M., Pinho C.S., Cott C.A. (2022). Person-Centered Rehabilitation Model: Framing the Concept and Practice of Person-Centered Adult Physical Rehabilitation Based on a Scoping Review and Thematic Analysis of the Literature. Arch. Phys. Med. Rehabil..

[41] Jesus T.S., Hoenig H. (2015). Postacute rehabilitation quality of care: Toward a shared conceptual framework. Arch. Phys. Med. Rehabil..

[42] Galvin K.T., Pound C., Cowdell F., Ellis-Hill C., Sloan C., Brooks S., Ersser S.J. (2020). A lifeworld theory-led action research process for humanizing services: Improving “what matters” to older people to enhance humanly sensitive care. Int. J. Qual. Stud. Health Well-Being.

[43] Terry G., Kayes N. (2020). Person centered care in neurorehabilitation: A secondary analysis. Disabil. Rehabil..

[44] MacLeod R., McPherson K.M. (2007). Care and compassion: Part of person-centred rehabilitation, inappropriate response or a forgotten art?. Disabil. Rehabil..

[45] Bender A.A., Burgess E.O. (2020). Constructing Recovery Narratives: Experiences and Expectations Following Spinal Cord Injury. Rehabil. Nurs..

[46] Plant S.E., Tyson S.F., Kirk S., Parsons J. (2016). What are the barriers and facilitators to goal-setting during rehabilitation for stroke and other acquired brain injuries? A systematic review and meta-synthesis. Clin. Rehabil..

[47] Gibson B.E., Terry G., Setchell J., Bright F.A., Cummins C., Kayes N.M. (2020). The micro-politics of caring: Tinkering with person-centered rehabilitation. Disabil. Rehabil..

[48] Jones F., Gombert K., Honey S., Cloud G., Harris R., Macdonald A., McKevitt C., Robert G., Clarke D. (2021). Addressing inactivity after stroke: The Collaborative Rehabilitation in Acute Stroke (CREATE) study. Int. J. Stroke.

[49] Killington M., Fyfe D., Patching A., Habib P., McNamara A., Kay R., Kochiyil V., Crotty M. (2019). Rehabilitation environments: Service users’ perspective. Health Expect..

[50] Clarke D., Gombert-Waldron K., Honey S., Cloud G., Harris R., Macdonald A., McKevitt C., Robert G., Jones F. (2021). Co-designing organisational improvements and interventions to increase inpatient activity in four stroke units in England: A mixed-methods process evaluation using normalisation process theory. BMJ Open.

[51] Sinclair L.B., Lingard L.A., Mohabeer R.N. (2009). What’s so great about rehabilitation teams? An ethnographic study of interprofessional collaboration in a rehabilitation unit. Arch. Phys. Med. Rehabil..

[52] Kayes N.M., Papadimitriou C. (2023). Reflecting on challenges and opportunities for the practice of person-centred rehabilitation. Clin. Rehabil..

[53] Barnett A., Ball L., Coppieters M.W., Morris N.R., Kendall E., Campbell K.L. (2023). Patients’ experiences with rehabilitation care: A qualitative study to inform patient-centred outcomes. Disabil. Rehabil..

[54] Hudak M.L., Graves A., Reichelt K.A., Sweigart J., Harry E., Glasheen J., Jones W., Cumbler E., Pelegano J.F., Wegman M. (2014). What makes a positive deviant: Utilizing common themes in best practice stroke hospitals to influence institutional quality improvement. Am. J. Med. Qual..

