# Supplementary material for: What Is So Great about Inpatient Rehabilitation from the Patient Experience Perspective: Qualitative Content Analysis of an Appreciative Inquiry during a Bedside Experience Rounding
Source: Healthcare (Basel). 2024 Aug 27;12(17):1711. doi: 10.3390/healthcare12171711 (PMC11394889; doi:10.3390/healthcare12171711)
Supplement: Supplementary file 1 [file healthcare-12-01711-s001.zip › healthcare-3093968-supplementary.pdf]

Supplementary Table S1: Coding structure.

Category	Subcategory	Codes
Staff attributes (interpersonal)	Kind, friendly, and smiling	Positive attitude; joyful; friendly; kind; cordial; smile of their face; upbeat; happy & energetic.
	Listening and responsive to	Listener; willing to listen; listens to and answer the questions; listens and tries to fix it; listens to and explains; asks and explains before doing; joy to talk with; detailed and/or helpful feedback; listens to me & makes adjusts or adapts to my preference.
	Encouraging (but not too hard) & reassuring	Encouraging; provides encouragement; tells me I'm doing or progressing nicely; inspire me to do better; reassure me when I am not in the best day (they listen but don't push); pushed but not forced; told me to give some time and see things becoming to work; when it does not go according to plan, they reassure me and it is ok; I am proud when they encourage and I make accomplishments – we celebrate together and I feel special; there is always something positive that I have done.
	Attentive and caring – beyond clinical duty	Helpful; genuinely caring; compassionate or understanding; really cares about me or my progress; I feel I have being taken care of; staff is always offering help; always available to talk; comes daily to check; open for a talk – beyond clinical duty; sits down and talks to me just to check in with me; we can talk about things other than medical stud; prompt to help and make you feel comfortable; paying attention to things I even not spoke about; never make you feel you are bothering them.
	Professional & Knowledgeable	Professionalism; professional; do their job professionally; competent; polite; expert; expertise; provide evidence-based care.
	Staff is nice, overall / unspecific	Great staff; terrific; amazing; pretty good; very nice; the whole staff was great; specific staff members were great; enjoyed the staff; doing what they can; like the staff; the staff member is good; work tirelessly; love the staff; who hires staff does a wonderful job.
Patient Care	Not Applicable	Enjoyed my therapy; liked my therapy; therapy has been great; workout is great; work in the gym; good work here; learning skills; work with my therapists and/or see my progress; variety of exercises; like when therapies are spaced out.
Leisure & social activities	Not Applicable	Socializing with others; outing with peers for recreation; going to the garden; going to the patio / outside; meeting new people and getting others' opinions - including in group therapy; having fun or doing fun things with peers – including in group therapy; interacting with peers in the gym; live music; attendings demonstrations; going around; go to the cafeteria; fun in the activities (e.g. making grilled cheese).
Built Environment	Not Applicable	Nice facility; high-tech facility; bright and cheerful; views are great; overall physical atmosphere.

Equipment	Not Applicable	Excellent gym's equipment; sufficient amount of gym's equipment – no need to compete for equipment; exciting to use all the equipment.
Teamwork	Not Applicable	Teamwork is good/great; practitioners help each other; one can tell there is communication between the team; every practitioner incorporates what I have been taught by others.
Cleanliness	Not Applicable	Cleanliness; facility is clean; clean and well-maintained
Food Services	Not Applicable	Food has been good; good meal selections.
Costumer Services	Not Applicable	Costumer services; movies on the tv.
Room Quality	Not Applicable	Pleasant room; lovely room.
Bed	Not Applicable	Beds are really great; bed is comfortable.
Quietness	Not Applicable	It is quiet.
